# Computer-Supported Meta-reflective Learning Model via mathematical word problem learning for training metacognition

**DOI:** 10.1186/s41039-018-0080-1

**Published:** 2018-09-25

**Authors:** Tama Duangnamol, Thepchai Supnithi, Gun Srijuntongsiri, Mitsuru Ikeda

**Affiliations:** 1 School of Knowledge Science, Japan Advanced Institute of Science and Technology, 1-1 Asahidai, Nomi, Ishikawa 9231211 Japan; 2 School of Information, Computer, and Communication Technology, Sirindhorn International Institute of Technology, Thammasat University, Pathum Thani, 12121 Thailand; 3 National Electronics and Computer Technology Center, Khlong Luang, Pathum Thani 12120 Thailand

**Keywords:** Computer-supported environment, Designing learning environment, Mathematical word problem, Metacognition, Self-regulated learners

## Abstract

To become a self-regulated learner, one needs to have a skill required to induce himself to comprehend their own cognition. In this paper, we provided a definition of Seed skill to become a self-regulated learner (S2SRL) as a basis terminology for developing our proposed framework, CREMA—Computer-Supported Meta-Reflective Learning Model via MWP in order to design an environment to encourage learners to use intrinsic comprehension of metacognitive questioning to acquire S2SRL in mathematical word problem (MWP) learning. To assess our proposed framework, we addressed these questions: (i) Can CREMA really support learner to gain S2SRL and (ii) How does it work in a practical environment? To answer these two questions, three classes of low performance students of grade 9 (total 101 students) were assigned into three different learning groups: (i) a group of students who learnt MWP with our proposed method by implementing CREMA, (ii) a group of students who learnt MWP in traditional method combining MetaQ—metacognitive questions and motivational statements, and (iii) a class of students who learnt MWP in traditional method. The result from our investigation showed that MetaQ played an important role in CREMA, while integrating computer and technology enhanced students’ learning sense and empowered methodology to facilitate learning objects in the implementation of CREMA to effectively support students to gain S2SRL in MWP learning.

## Introduction

Transforming learners to become self-regulated lies at the heart of education. After school or university, students face problems in their daily life that can be overcome provided that they have mastered the skill needed to solve the problems on their own. Zimmerman (2002) defined self-regulated learners as those who are motivated to automatically perform monitoring and regulating their learning processes and be aware of their learning difficulties to achieve their tasks; in other words, self-regulated learners must have motivation for maintaining their emotion/behavior to perform metacognitive skills or to (either implicitly or explicitly) perform metacognitive questioning to reflect their own cognition to do planning, monitoring, and self-evaluation to accomplish their tasks.

However, training metacognition is not a simple task due to the implicitness of metacognition and the complication of its training process. In particular, to motivate learners to perform metacognitive skill or to transform their learning status from passive to self-regulated is a challenge. According to the OECD report (2010), explicit or formal instruction of metacognitive strategies leads to an improvement in students’ learning performance. It showed that students who received cognitive and metacognitive strategy instruction made more significant gains on measures of reading comprehension than students who are only trained with conventional instruction (Baker and Carter-Beall 2009; Dole et al. 2009; Waters and Schneider 2010). However, to perform meta-level thinking or to do self-reflection by metacognitive questioning is a daunting task for young or novice learners who have never been trained or been familiar with this kind of activities. Therefore, in this research, we aim to develop a framework to design a learning environment to promote and support their meta-level thinking skills.

To avoid producing cognitive load and frustration in metacognitive training, which might cause demotivation in novices, and to encourage learners to become familiar with and be able to perform metacognitive skill, we believe that there should be an implicit meta-level thinking skill, a basic skill that serves as an assisting ladder that enables them to develop themselves so as to fully become self-regulated learners. We have named that implicit skill as Seed skill TO become Self-Regulated Learners (S2SRL). Here, S2SRL is defined as a skill in which learners are curious about their understanding and are aware of their self-improvement in the learning before they can perform metacognitive questions on their own, and in so doing, they can reflect on their cognition for planning, monitoring, and doing self-evaluation. The terminology is so defined with a view to developing and improving our required framework.

As mentioned earlier, it is a difficult task for novices to think about metacognitive questions by themselves without having experience. Therefore, in this study, instead of simply encouraging novices to perform metacognitive questioning, it should be helpful if there is an environment to engage and encourage learners to perform intrinsic comprehension of metacognitive questioning so that they can acquire S2SRL, eventually preparing them for the next step of metacognitive training. To encourage learners to gain S2SRL, it is necessary to motivate and facilitate them to clarify their own cognitive process of a given task in their mind. Later, they can use the experiences they have gained and stored in their minds as long-term memories as their cognitive target to perform meta-level thinking (Kayashima et al. 2005).

According to Livingston (2003), cognitive strategies are used to help a learner achieve a goal while metacognitive strategies are used to ensure that the goal has been reached, that is, learners cannot perform meta-level thinking without base-level activities or cognitive strategies. In this study, mathematics is considered as a medium for performing cognition because it is a compulsory subject in both elementary and secondary levels of education in all countries. The topic in mathematics that we choose is an algebraic approach to solve Mathematical Word Problem (MWP)—mathematical problems written in context in which students learn to model a problem described in natural language into mathematical notation—because it is a simplest application in mathematics that links an abstract concept to a real-world application. A bitter pill for most students, MWP, however, provides a room to apply meta-level thinking in its solving process. The main difficulty that students encounter in solving MWP is to construct a problem model by making inferences from the problem context (Fuchs et al. 2008; Jacobse and Harskamp 2009). It was revealed by Schoenfeld (1992) that the difficulty arises because they seldom spend time on monitoring and regulating the use of their own cognitive strategies. This causes them to omit or put a wrong interpretation on information from the problem and misleads them to make an inappropriate decision on choosing a solution (Verschaffel et al. 1999). Moreover, there are studies which have found a strong association between reading proficiency and metacognition (Artelt et al. 2001; Brown et al. 2004) particularly through MWP solving because it involves a process to practice reading comprehension. Moreover, MWP solving has “explicit form of solution process” which is a good feature to support monitoring and to create representation framework to externalize problem-solving process. And its “complexity of solution process” and “many explicit operators at each step” are beneficial features to support metacognitive training in which the former feature promotes reflective analysis of the thinking process, while the latter feature helps promote regulation of decision making criteria. These are considered as advantage features of MWP, which can be employed as a medium to practice meta-level thinking.

Since there are a number of students in a class and individual students are different, adaptive environment should play a role in this situation. To promote metacognitive questioning corresponding with the learners’ learning process in an adaptive way together with various kinds of representation/media to support and facilitate the learning process, computer technology is considered for this role. Research shows the potential of using computer technology to support self-regulated learning in which a new and promising research subject may be assessing the effects of computer environments, which combine cognitive content with metacognitive support or as a construction tool for creating representations of mental models, for example, by using intelligent tutoring systems, educational multimedia systems, virtual agents, metacognitive hints, and so on (e.g., Jacobse and Harskamp 2009; Nakano et al. 2002; Schraw et al. 2006). To achieve our desire to have an environment for encouraging learners to use intrinsic comprehension of metacognitive questioning to acquire S2SRL in MWP learning, instead of proposing a particular environment, we have developed the so-called Computer-Supported Meta-Reflective Learning Model via Mathematical word problem learning (CREMA) to be a framework for designing such an environment.

The rest of this paper gives more detail on background theories to define S2SRL in MWP learning as a basis terminology for developing the proposed framework, CREMA; then, the learning architecture of CREMA is revealed. Crucially, the methodology to validate the proposed model is analyzed and discussed from its empirical result before a final conclusion is made.

## Defining S2SRL in MWP learning

In this section, we provide related theories to illustrate how S2SRL in MWP learning is defined and to prepare a tool for assessing our proposed framework.

### The role of cognition, metacognition, and motivation in self-regulation

**Fig. 1:**
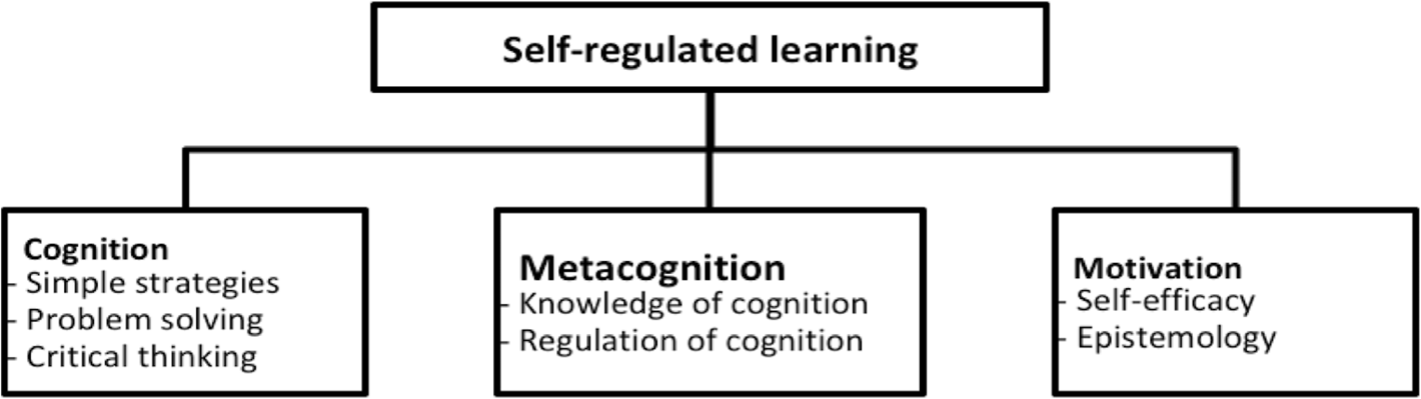
Components of self-regulated learning (Schraw et al. 2006)

According to the self-regulating model proposed by Schraw et al. (2006), self-regulated learning consists of three main components (each component could be divided into subcomponents): cognition, metacognition, and motivation (Fig. 1).

Learners’ motivation may come from their goal orientations, attitudes, self-efficacy beliefs, expectations, social sources, helpfulness, moral principle, and interests (Schunk 2008; Zimmerman 2008). The term “motivation” refers to any kind of ordinary ambition for doing something (Baumeister and Vohs 2007). Motivation includes self-efficacy and epistemological beliefs that affect the use and development of cognitive and metacognitive skills. As mentioned earlier, learners use cognitive strategies to achieve a goal, while they use metacognitive strategies to make sure that the goal has been reached (Livingston 2003). Cognition includes three types of learning skills: cognitive strategies, problem-solving strategies, and critical thinking skills, enabling learners to encode, memorize, and recall information. Metacognition enables us to become successful learners and has been associated with intelligence. It is higher order thinking, which involves active control over the cognitive processes engaged in learning. It includes two main components: knowledge of cognition and regulation of cognition, enabling learners to understand and monitor their cognitive processes. Metacognitive knowledge of cognition refers to the knowledge about cognitive processes, one that can be used to control cognitive processes. It can be divided into knowledge of person, task, and strategy variables. Metacognitive regulation of cognition involves the use of cognitive strategies or cognitive regulation.

**Fig. 2:**
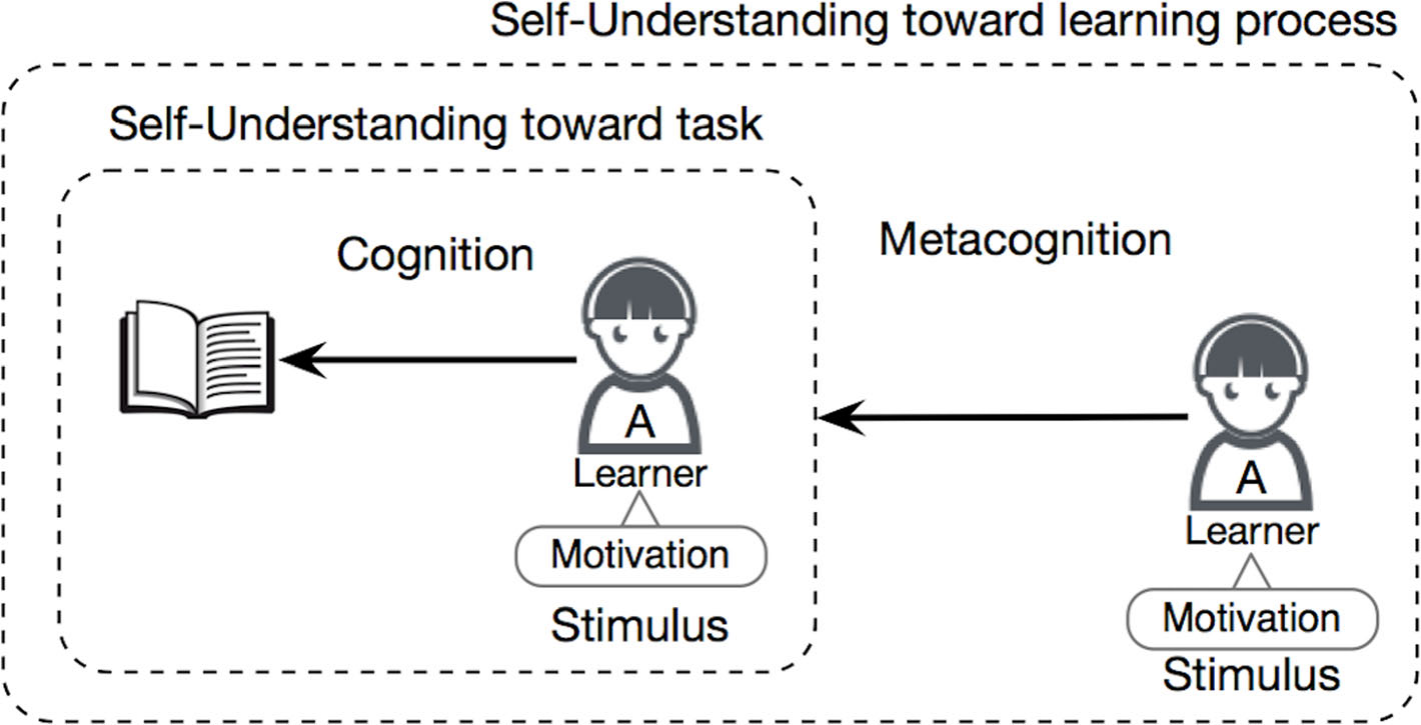
Relation of cognition, metacognition, and motivation in self-regulation

To perform metacognition, learners should be able to have clear understanding of their cognition in which their motivation plays an important role in this self-regulation as stimulus to stimulate their cognitive and metacognitive strategies. Therefore, in this dissertation, we consider required skills of self-regulated learners in three aspects: stimulus, self-understanding toward task, and self-understanding toward learning process, as illustrated in Fig. 2. The detailed explanation of each aspect is described in the following section.

### Required skills for self-regulated learners

#### Learning stimulus

It is necessary that self-regulated learners have skills to stimulate and drive their learning desire. What we consider as learning stimulus here are attitude adjustment, goal setting, and motivation management.

##### Attitude adjustment

Research showed that attitude is one of the most crucial factors that can predict academic achievement. Positive attitude to learn is not inborn—it requires time and effort to be developed and encouraged (Credé and Kuncel 2008). If learners have confident attitudes and perceptions, they have a mental climate—a function of the attitudes and perceptions of learners—that is good for learning. If those attitudes and perceptions are not in place, learners have a mental climate unsuited for learning (Marzano 1992). It is important that learners realize their own feeling and thought to make themselves feel easy in learning MWP. But, on the emotional level, learners might be struggling: they may think MWP is too difficult for them or they feel that they cannot do it. In order to be successful in any kinds of tasks, it is essential to develop a good attitude in learning those tasks.

##### Goal setting

Learning goal is thought to be a guideline to regulate learners’ learning behaviors (Schunk 2001). Encouraging learners to set short-term goals can also be an effective method to support them to keep track of their learning progress (Zimmerman 2004). To reach long-term ambition, short-term achievable goals are helpful. For instance, if a learner set their long-term goal to get an A in mathematics, they may set their achievable goals such as submitting all assignments and attending every class as well as paying close attention to the teacher, which will help them understand the difficult topics better and would eventually bring them within achievable reach of a top grade.

##### Motivation management

When learners autonomously apply strategies to keep track of achieving a learning goal, motivation occurs. Motivation is essential to self-regulated learning process by virtue that it requires learners to presume regulation over their cognition (Corno 1993). According to Zimmerman (2004), motivation is found in the absence of external rewards or stimulus. As a consequence, it can be a crucial indicator that learners will become more independent. Learners are able to withstand difficult tasks and frequently feel better and more pleasant with the learning process when they set their own learning goals and find motivation from within to make progress toward those goals (Wolters 2003). Therefore, encouraging learners to find motivation in learning MWP would help them to improve their self-regulation.

#### Self-understanding toward task

To become a self-regulated learner in MWP, it is important that a learner should (i) understand their MWP background knowledge, (ii) understand their self-understanding of MWP principle, and (iii) understand their self-difficulties in MWP learning to be able to improve their MWP learning performance and master the task.

##### Background knowledge understanding

Supporting learners to observe their own background knowledge on the topic that they have to learn helps them to gain more understanding on their own about what they know and what they do not toward the topic that would help them in planning. Schunk (2001) indicated that planning and goal setting are processes, which work harmoniously. Planning could assist learners in shaping well thought-out goals and strategies to achieve a task. He expressed that planning involved in three stages of learning process: setting a goal, forming learning strategies to accomplish the goal, and verifying the possibility to achieve the goal. There have been studies (e.g., Pressley 1990; Scheid 1993), which showed evidence that teaching learners to catch up learning tasks by performing planning is a practical way to promote self-regulation. Therefore, in order to help learners to perform good planning to ensure achievement toward a given task, encouraging or supporting them to realize and understand their background knowledge would be helpful.

##### Self-understanding of principle of topic

Learners must set learning goals, make a plan, motivate themselves to achieve the goals, keep their focus on the assigned task, and adjust their learning strategies to acquire comprehension of learning material, in order to monitor their own learning progress (Zimmerman 2004). It is necessary that learners presume ownership for their learning and attainment results in order to develop themselves to be strategic learners (Kistner et al. 2010). Complementarily, monitoring one’s own learning process helps learners to better understand their own cognition. Encouraging learners to be curious about their own understanding of the principle of solving MWP helps them to monitor their learning process.

##### Self-difficulty understanding

Winne (2009) revealed that learners likely become self-regulated learners when they have abilities to evaluate their own learning and are able to be independent of summative assessments in their learning class. Learners who can evaluate their learning can understand more about their own learning difficulties. This may facilitate them to make adjustment for next similar tasks (Schraw and Moshman 1995). Ryan et al. (2001) elaborated that the difference between self-regulated learners and their peers is that they do not only seek advice from others, but they do so with the goal of making themselves able to rely on their own. If learners precisely understand their learning difficulties, it enables them to find appropriate methods or to effectively seek proper help to accomplish the assigned tasks.

#### Self-understanding toward learning process

Self-understanding toward learning process is composed of (i) understanding of their MWP learning strategy—they need to monitor, regulate, and alter their learning strategy—and (ii) understanding of their learning concentration of the topic to be able to achieve their learning goal.

##### Learning strategy understanding

The ability to implement multiple learning strategies across tasks and modify those strategies as required is essential for self-regulated learners to facilitate their progress toward their expected goals (Paris and Paris 2001). However, for novice or less experienced learners, it might be very difficult to think about various strategies as options. As revealed in Van den Broek et al. (2001), most primary grade students in their study did not have a large repertoire of learning strategies at their disposal. Therefore, appropriate amount of examples and scaffolding would help learners to gain more experience and acquire more skills to be able to perform the task by themselves. Encouraging learners to be curious about their own learning strategies would support them to pursue their learning goal.

##### Learning concentration understanding

In order to support learners to keep focus on their learning process and not to be distracted before achieving their goals, maintaining self-concentration in the learning process is also important. Self-regulated learners must be able to control their attention (Winne 2009). There is research that showed that academic performance positively correlated with focused time spent on tasks (Kuhl 1985). Frequently, attention control refers to ridding of learning distraction in one’s mind, together with making or finding surrounding environment to be conducive to learning (Winne 2009). Therefore, it is crucial to encourage learners to be curious about their sources of learning distractions so that they can find a way to resolve the distractions and build up their learning concentration to expand their attention spans.

### S2SRL in MWP learning

The interview and survey were conducted as qualitative and quantitative confirmation for the viability of our proposed required skills of self-regulated learners in MWP learning based on previous research and related theories in the previous section.

In the interview, ten students who were self-regulated learners in mathematics participated. They were from three different schools in Thailand. Most of them were reported by their mathematics teachers to be academically outstanding and highly responsible, working on their assignments themselves and submitting them in time, and were known to participate actively in their mathematics classes. The brief summary from the interviews is shown in Appendix 1. By the theoretical review and the interview, we summarize the required skills of self-regulated learners in MWP learning in Table 1.

**Table 1:** Required skills of self-regulated learners in MWP learning for this dissertation

Aspects	Categories	Explanation
Stimulus	Attitude	I am curious about the source of my feeling and think about how to find the benefit/application of learning MWP to make it easy for me to learn MWP.
	Goal	I am curious about my goal of MWP learning and think about how to encourage myself to achieve the goal I set for learning MWP.
	Motivation	I am curious about my reason why I should have to learn MWP to motivate myself in accomplishing my goal.
Self-understanding toward task	Background knowledge	I am curious about what I know in learning MWP and also curious to find a way to update my background knowledge to meet the knowledge required for learning MWP.
	Self-understanding of principle of topic	I am curious about my understanding of MWP principle and also curious to find method to improve my understanding of MWP principle.
	Self-difficulty	I am curious about my difficulty in MWP learning and always think about the way to resolve it to be able to improve my performance.
Self-understanding toward learning process	Strategy	I am curious about the appropriate strategy to achieve my goal in MWP learning and always think about finding my own effective way to achieve my goal in MWP learning.
	Concentration	I am curious about the source of my distraction in learning MWP and want to find a way to resolve it so that I can concentrate on my learning.

The eight statements in Table 1 were used in the survey. We asked participants to rate their confidence as self-regulated learners (0–10 confident interval, from not confident at all to very confident, respectively) and then asked them to rate how much they agree or disagree with each of the eight statements (1—very untrue of me, 2—untrue of me, 3—somewhat untrue of me, 4—neutral, 5—somewhat true of me, 6—true of me, and 7—very true of me). The participants were students of grades 8–12 from both public and private schools in Thailand who have already learnt MWP. In total, there were 699 students from 31 schools who responded to the survey.

A Pearson correlation coefficient was computed to assess the relationship between level of confidence as self-regulated learners and level of each proposed required skill. There was positive correlation between the two variables for all eight items, *r*s(699) > 0.6, *p*s < 0.001. An independent-sample *t* test was conducted to compare two groups of participants who were confident as self-regulated learners in learning MWP (SR: the participants whose self-reported level of confidence was at least 7, there were 247 participants in this group) and who were confident as non-self-regulated learners in learning MWP (nonSR: the participants whose self-reported level of confidence was not above 3, there were 125 participants in this group) for all eight items. The analysis of the result shows that, for all items, there were significant differences in the scores of SR and nonSR, *t*s(370) > 16, *p*s < 0.001, as shown in Appendix 2.

In summary, the quantitative statistical analysis from the survey implies that self-regulated learners in learning MWP have a strong tendency to have the proposed skills. By the qualitative analysis from the interview, it could explain the phenomenon that for those who really like mathematics, distraction was not a problem for them because they learned it with passion and mathematics was their first priority; however, for the self-regulated who might not enjoy mathematics as much, they were much more concerned with getting rid of learning distraction. These items would be modified as a questionnaire for classifying a learner who gained S2SRL in MWP learning (Q-L2SRL) for the later investigation.

Through the theoretical review which was later confirmed by the qualitative and quantitative study, we define S2SRL in MWP learning as a basis skill that learners can further develop to gain the required skills of self-regulated learners in MWP learning, that is, learners are curious about their own “understanding of MWP learning” and have “awareness of self-improvement in MWP learning” before they can perform metacognitive questions by themselves to reflect on their own cognition for planning, monitoring, and doing self-evaluation. “Understanding of MWP learning” and “awareness of self-improvement in MWP learning,” here, are considered in three aspects: stimulus, self-understanding toward task, self-understanding toward learning process, as shown in Table 2.

**Table 2:** Categories in each aspect of UL and ASL

Aspects	Categories	Understanding of MWP learning (UL)	Awareness of self-improvement in MWP learning (ASL)
Stimulus (STM)	Attitude (STM-A)	Understanding of their attitude on MWP learning	Awareness of self-improvement in their attitude on MWP learning
	Goal (STM-G)	Understanding of their goal on MWP learning	Awareness of self-improvement in their goal on MWP learning
	Motivation (STM-M)	Understanding of their motivation on MWP learning	Awareness of self-improvement in their motivation on MWP learning
Self-understanding toward task (SUT)	Background knowledge (SUT-K)	Understanding of their MWP background knowledge	Awareness of self-improvement in their MWP background knowledge
	Self-understanding of principle of a topic (SUT-P)	Understanding of self-understanding of MWP principle	Awareness of self-improvement in self-understanding of MWP principle
	Self-difficulty (SUT-D)	Understanding of self-difficulty in MWP learning	Awareness of self-improvement in self-difficulty in MWP learning
Self-understanding toward learning process (SUP)	Strategy (SUP-S)	Understanding of their strategy of MWP learning	Awareness of self-improvement in their strategy of MWP learning
	Learning Concentration (SUP-C)	Understanding of their concentration of MWP learning	Awareness of self-improvement in their concentration of MWP learning

## CREMA

**Fig. 3:**
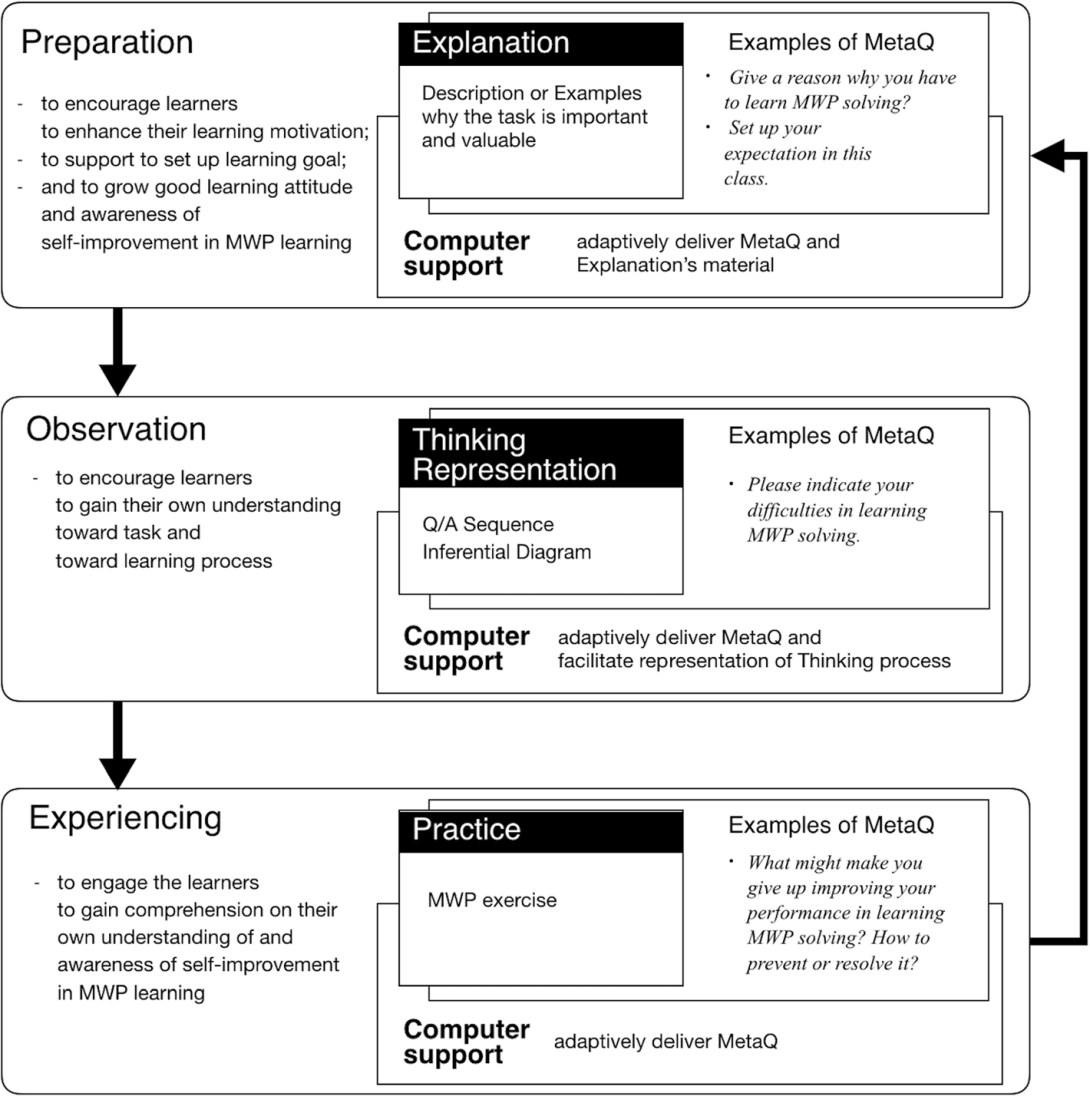
Diagram of the learning cycle of CREMA

In this research, CREMA is proposed as a framework for designing a learning environment encouraging learners to use intrinsic comprehension of metacognitive questioning to acquire S2SRL in MWP learning. The design intention of a learning environment implemented using CREMA as a framework is to support/facilitate learners to learn how to learn MWP and get used to performing self-reflection on meta-level thinking in MWP learning by using technology that enhances their learning sense and empowers methodology to facilitate learning objects. We designed CREMA as a holistic approach to provide support related to required skills. Figure 3 illustrates the structure of CREMA. It is represented into three phases to support required skills: Preparation phase, Observation phase, and Experiencing phase. Each phase in the diagram shows the target skills and the kinds of learning support involved. For example, in Preparation phase, metacognitive questions and motivational statements (MetaQ) are applied with Explanation—description or examples why the task is important and valuable—to encourage learners to enhance their motivation, to support their learning goal creation, and to grow their good learning attitude as well as their awareness of self-improvement in MWP learning.

The detailed explanation and demonstration of each phase and its support theories are described in the following subsections.

### Preparation phase

When learners are motivated to learn, they are more likely to spend time and effort on the learning task and apply self-regulated learning skills, and when they can successfully utilize self-regulation strategies, they are more motivated to accomplish learning tasks (Zimmerman 2000). They involve their interests and values in making a decision, when they contemplate why they should complete the task and how hard it is. If they do not think a learning task is important enough, they are less likely to take time in setting goals and planning to accomplish the task (Simons et al. 2004; Wang and Holcombe 2010; Wolters 2003). As shown in Fig. 3, in this phase, MetaQ is integrated with Explanation. Explanation here refers to description or examples why the task is of importance and value. It is important that learners have a positive attitude toward and motivation to do their tasks. Then, in this phase, MetaQ and Explanation are applied in order to prepare learners’ mental readiness for the learning process. An environment of Preparation phase for its investigation (for evaluating CREMA, in the next section) is demonstrated as follows.

#### Environment of Preparation phase for its investigation

**Fig. 4:**
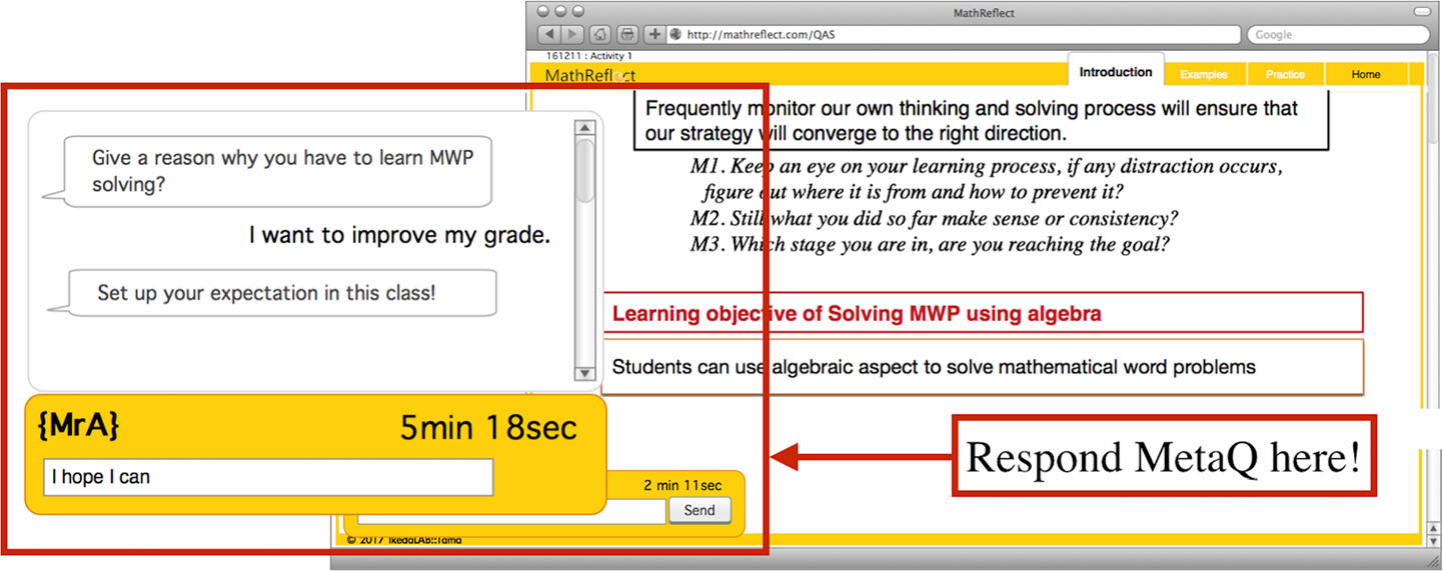
MetaQ raised in the introduction page of MathReflect

This phase included an extra period (taking place prior to the class). In the extra period, the teacher explained how important it was to learn MWP and gave some examples of applications of MWP in daily life. The teacher asked the students about their dream job and gave certain MWP application examples. Then, the teacher told the students to write down their goals for learning MWP and asked them to think about their motivation that would drive them to achieve their goals. Then, in the first period of the course, the students were allowed to gain access to the system, called MethReflect modified from (Duangnamol et al. 2017). The scope of the Preparation phase in MathReflect was only on the introduction page—the first page that greeted the students once they started the activity in the system upon logging in. In the introduction page, the learning objectives of the training program and the topic were provided. There was a direction informing the students to read and gain an understanding of the provided information. The students could move on from this page or this phase only after they had responded to MetaQ from the system in the dialog box in the bottom left of the page, (Fig. 4). MetaQ raised in this page were “Q1: Give a reason why you have to learn MWP solving? Q2: Set up your expectation in this class”*.* The system provided examples of answers of the MetaQ (*Example answers of Q1: I want to be good at MWP solving, To use it for my career, I want to be an engineer, I want to improve my grade, I want to graduate with a good grade, I want to make my parents proud of me, I want to be able enter into a good study program at university,* etc.*; Example answers of Q2, can understand more about MWP. can interpret context problem into math notation, can apply MWP in daily life problem,* etc.) and suggested the students to choose or use their own opinions.

### Observation phase

In this phase, we aim to encourage learners to gain self-understanding toward task and learning process in MWP learning, which supports them to increase self-efficacy belief. Zimmerman (2000) revealed that self-efficacy belief plays an important role in self-regulation. Increasing self-efficacy beliefs has positive impact on the use of self-regulation strategies (Bouffard-Bouchard et al. 1991; Pajares 2008; Schunk 1984).

**Fig. 5:**
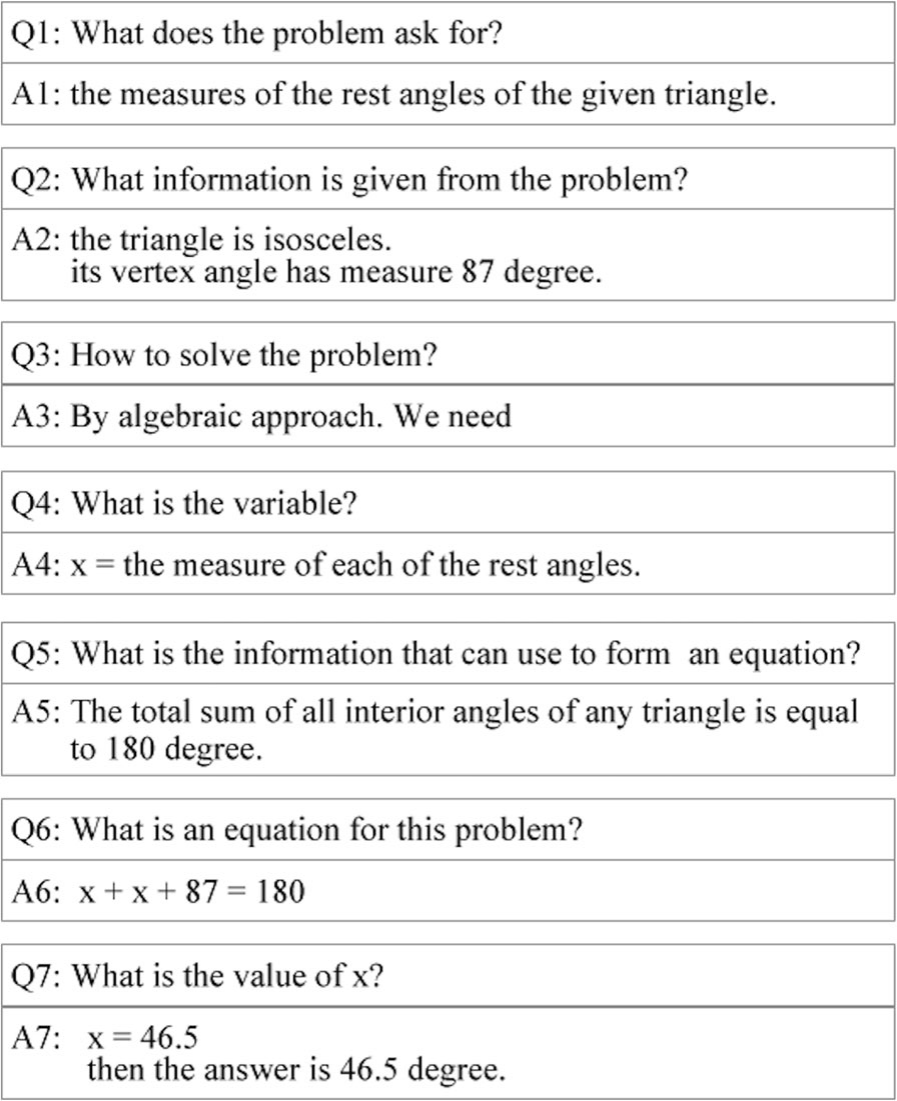
An example of QAS. It is a sequence of questions and answers to acquire information on how to accomplish a solution of a given MWP. InDi is a diagram showing a flow of information and its source/reason to be composed for accomplishing a solution of a given MWP

**Fig. 6:**
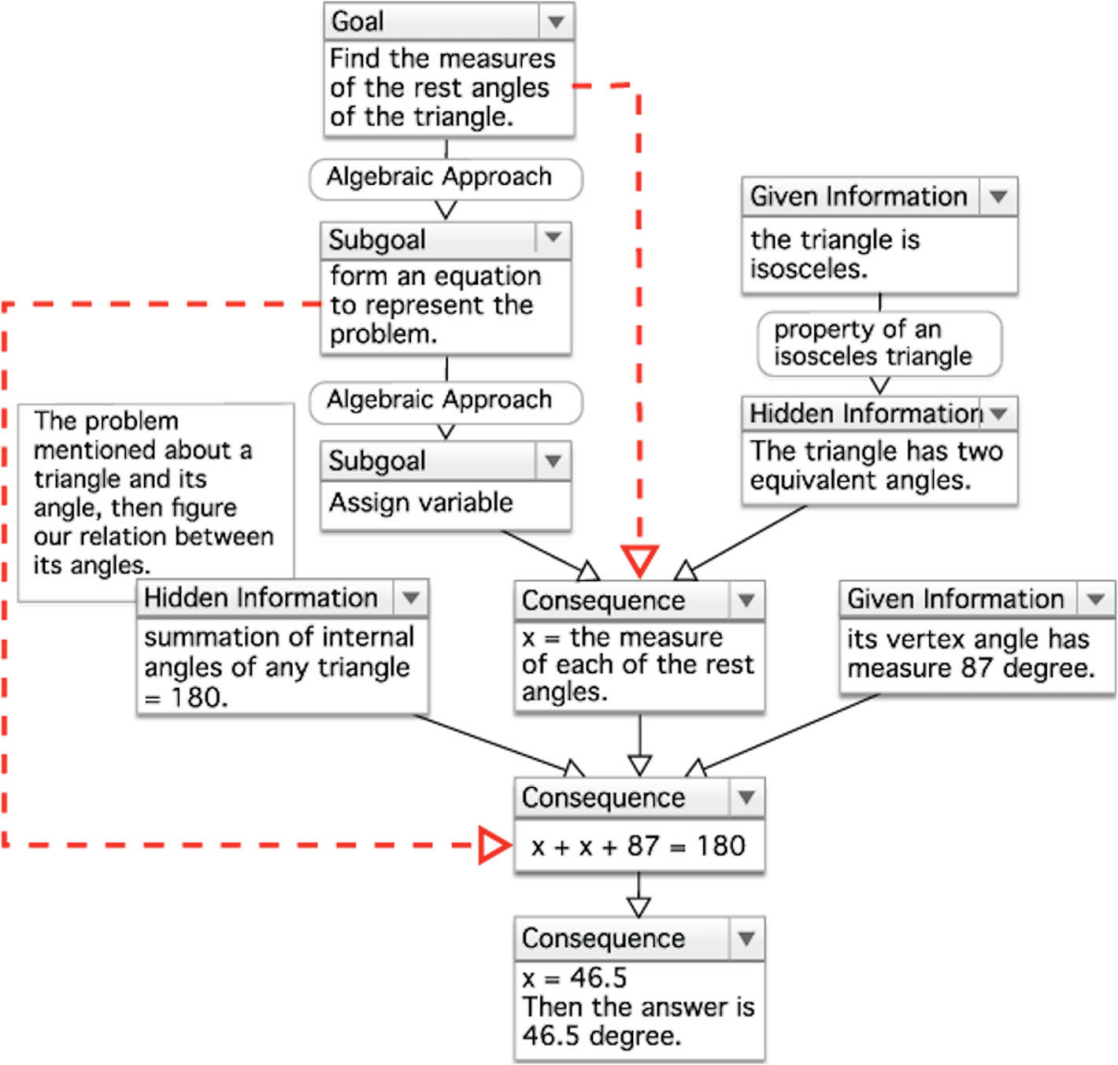
An example of InDi. It is composed of Information Node (in a rectangle)—to show information required, Information Tag (in top of each Information node)—to indicate the source of the information (there are six tag options: Goal, Sub-Goal, Given Information, Hidden Information, Result, and Others), Order Link (solid arrow)—to show consecutive order in which the information is used, Reason (in a rounded-corner rectangle over certain Information nodes)—to indicate why information is applied, and Sequential Link (dashed arrow)—to illustrate the result which needs information that is not consecutively linked

Externalizing thinking process into an observable format helps learners to reduce their cognitive load and enables them to observe and reflect on their thinking process more easily (Kayashima et al. 2005). This corresponds to the study of Rau et al. (2015) which showed that multiple external representations could significantly enhance learners’ learning. To achieve the aim of this phase, thinking process of MWP solving is simulated as Q/A sequence (QAS; see Fig. 5) and Inferential Diagram (InDi; see Fig. 6) to facilitate learners to observe their thinking process of MWP solving and to understand more clearly their MWP learning (Duangnamol et al. 2015). Consequently, MetaQ is applied to enable them to engage in reflecting on their own understanding of task and learning strategies of MWP learning by the support of QAS and InDi. An environment of the Observation phase for its investigation (for evaluating CREMA, in the next section) is demonstrated as follows.

*Use algebra to solve a given MWP* (MWP for Figs. 5 and 6).

A measure of a vertex angle of an isosceles triangle is 87°. What are the measures of the rest angles of this triangle?

#### Environment of observation phase for its investigation

MathReflect was applied until this phase. After the students had answered MetaQ in the previous page, they could access the Observation phase. The activities/tasks in this phase were composing QAS, completing InDi, and answering MetaQ. Once the students had entered the first page of this phase, MWP was shown with the direction informing them to read the problem carefully. Then, a question was raised, “Do you completely understand the problem?” They could respond to this question by clicking on the buttons, YES or NO. If they went for YES, QAS constructing page appeared; otherwise, the list of possibilities of difficulties (e.g. *do not know the meanings of some words in the problem, cannot imagine the situation in regard to the problem, do not understand the situation in the problem*) was suggested as examples together with the direction for telling them to answer MetaQ. MetaQ raised here was “What do you think it is the reason that you cannot understand the problem clearly? (Choose from the list or state your own opinion)”. They could only move to QAS constructing page only if they had finished answering MetaQ.

**Fig. 7:**
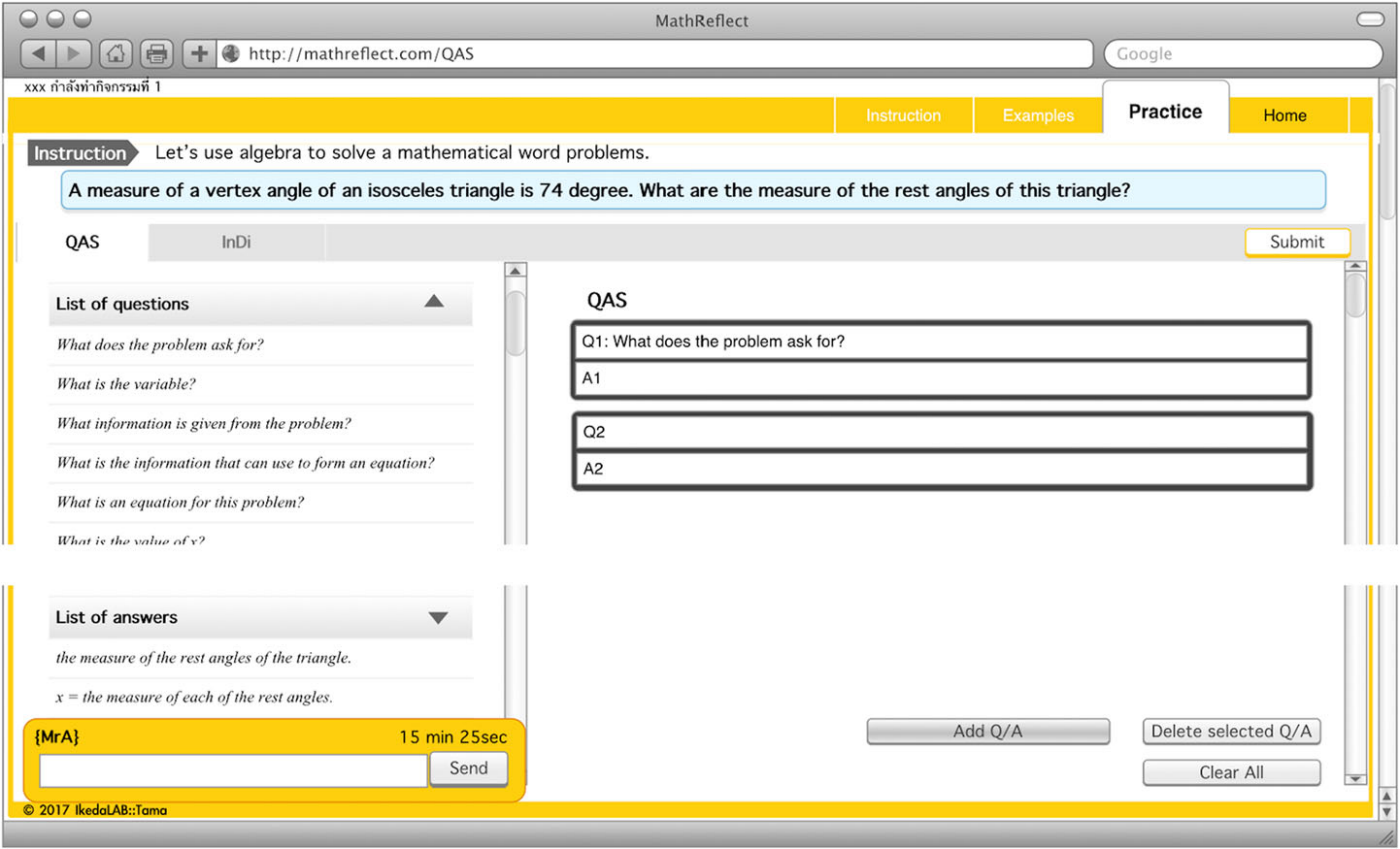
Web interface of MathReflect at QAS constructing page

**Fig. 8:**
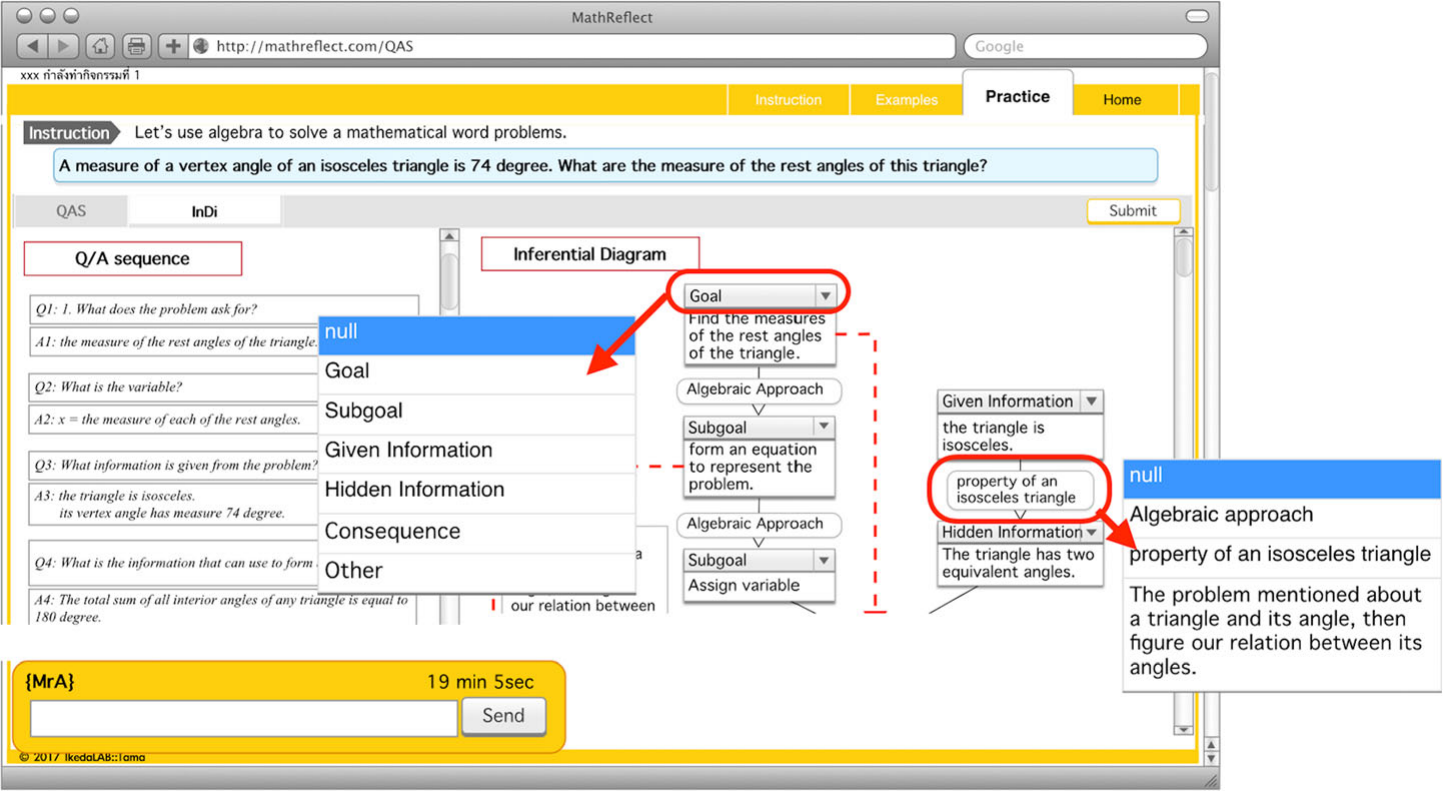
Web interface of MathReflect at InDi completing page

- *Procedure in QAS constructing page* (Fig. 7): Students had a task to match questions and answers; then, put them in an appropriate order (Fig. 6). If they had composed it correctly, they could proceed to answering the MetaQ, “What is your problem to compose QAS? Or Which question might be difficult for you?”, and be given permission to access the next page. Conversely, incorrect QAS left them with no permission to access the next page. The system could suggest that their ongoing QAS had wrong pair of Q/A or unreasonable sequence when they submitted incorrect QAS. If the students believed they could not do it, they could click for a hint to see a solution and follow it; however, this action would be recorded and prevented them from moving over from this task. If the class period was over before they finish the task, unfortunately, they needed to start to compose that QAS from the beginning next time they logged-in into the system.
- *Procedure in InDi completing page* (Fig. 8): The students were tasked with selecting appropriate Information Tags and Reasons from the provided list of existing information to make InDi complete and respond to MetaQ. In the same manner as on the QAS constructing page, the students could move over this page for the next step only if they had completed InDi correctly and answered MetaQ, “Which #box of information is difficult to remember?”

The students could finish the Observation phase only if they had composed QAS and completed InDi without following a solution suggested from the system. The same problem re-occurred until they could solve it themselves. Then, a new problem was shown until they were able to complete an unseen problem without clicking for a solution. The system then delivered these MetaQ’s:

- Evaluate your competency in solving MWP from observing this QAS and InDi as, poor, average, or excellent
- Please indicate your difficulties in learning MWP solving (Choose from the above list or state your own opinion)
- What might make you give up improving your performance in learning MWP solving? How can one prevent or resolve it?

The students needed to answer all questions to complete the phase. The number of the periods that the students took to finish this phase varied depending on individual performance.

### Experiencing phase

In this phase, the learners should have time to experience/internalize what they have learnt from the previous phases. MetaQ is applied while they are practicing solving MWP. To do this, a MWP solving task is assigned to the learners concurrently with asking them to predict and evaluate their performance both before and after. The learners have the situation to evaluate their performance on the learning task with respect to the effectiveness of the strategies that they choose. During this stage, the learners have a chance to manage their emotions about the outcomes of their learning experience. These self-reflections then influence their future planning and goals, initiating the cycle to begin again. Moreover, they can reflect on their performance in this phase and monitor their difficulties in learning MWP to engage them to reflect on what and how they can improve themselves to master in the topic. An environment of Experiencing phase for its investigation (for evaluating CREMA, in the next section) is demonstrated as follows.

#### Environment of experiencing phase for its investigation

Google Classroom was used in this phase. The students access Google Classroom to do MWP exercise while they could also access MathReflect any time to see their completed QAS’s and InDi’s. The students had to answer MetaQ before (*Read the question carefully, evaluate your confidence to complete this problem as percentage, before writing a solution*) and after (*After your attempt, evaluate your solution in percentage of completion*) solving MWP. After they had finished a few problems, they were asked to respond to these MetaQ’s;

- Critique your own difficulties in learning MWP solving.
- What might make you give up improving your performance in learning MWP solving? How can one prevent or resolve it?

After each period, the students got an assignment to complete an exercise on the MWP topic with which they had stated they struggled. The exercises were prepared in Google Classroom with solutions for several levels of performance. The teacher played a role of a supporter when the students needed more explanation.

To investigate the effects and conditions of CREMA more deeply in practice, we performed the experiment as described in the next section.

## Evaluating CREMA

In this section, we aim to investigate our framework by answering the following questions:

1. Can CREMA really support learners to gain S2SRL in MWP learning?
2. How does CREMA work in practical environment? (This question is considered in these following sub-questions)
  2.1 Is MetaQ a factor in CREMA to support learners to gain S2SRL?
  2.2 Can computer support really enhance training effect in CREMA?

### Methodology

To answer the first question, a group of students who had learnt MWP with the proposed method by implementing CREMA was compared with another group of students who had also learnt MWP but in a traditional way. To answer the second question, we had considered two sub-questions. In question 2.1, a group of students who had learnt MWP in a traditional way was compared with another group of students who had learnt MWP also in a traditional way but combined with MetaQ to investigate and ensure the effect of the intervention of MetaQ in a traditional class. In question 2.2, a group of students who had learnt MWP with the proposed method by implementing CREMA was compared with another group of students who had learnt MWP in a traditional way combined with MetaQ to see the effect of using MetaQ with and without computer support from implementing CREMA. In summary, these following groups of students were considered:

1. *Control Group 1* (**CTRL**): Students in this group had learnt MWP solving in a traditional way.
2. *Control Group 2* (**CTRL+MetaQ**): Students in this group had learnt MWP solving in a traditional way combined with the intervention of metacognitive questioning and motivational statements, their learning environment was the same with the **CTRL** group as shown in Fig. 9.

**Fig. 9:**
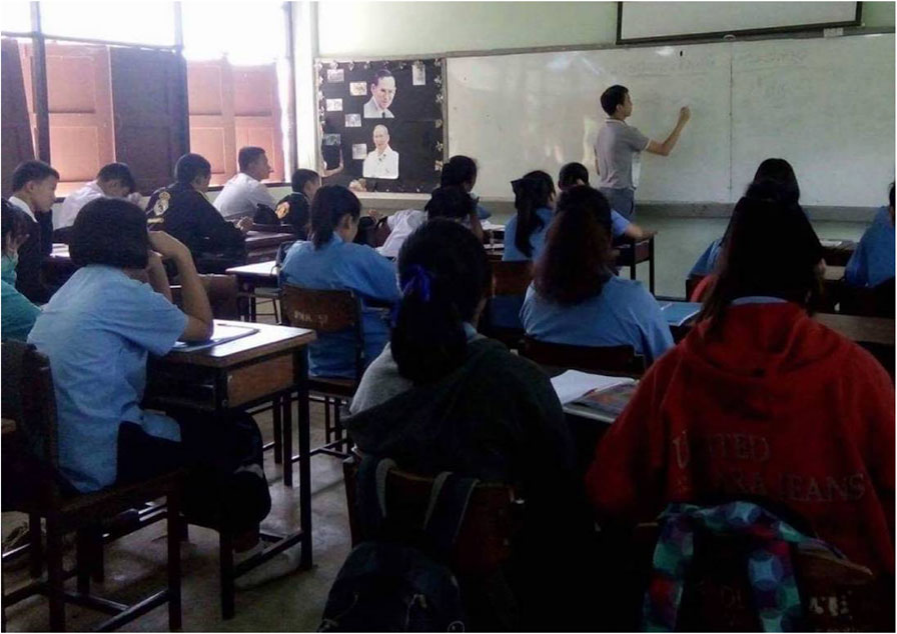
Learning environment of CTRL and CTRL+MetaQ
3. *Experimental Group* (**CREMA**): Students in this group had learnt MWP solving via computer application implemented through the application of CREMA as a framework, see Fig. 10.

**Fig. 10:**
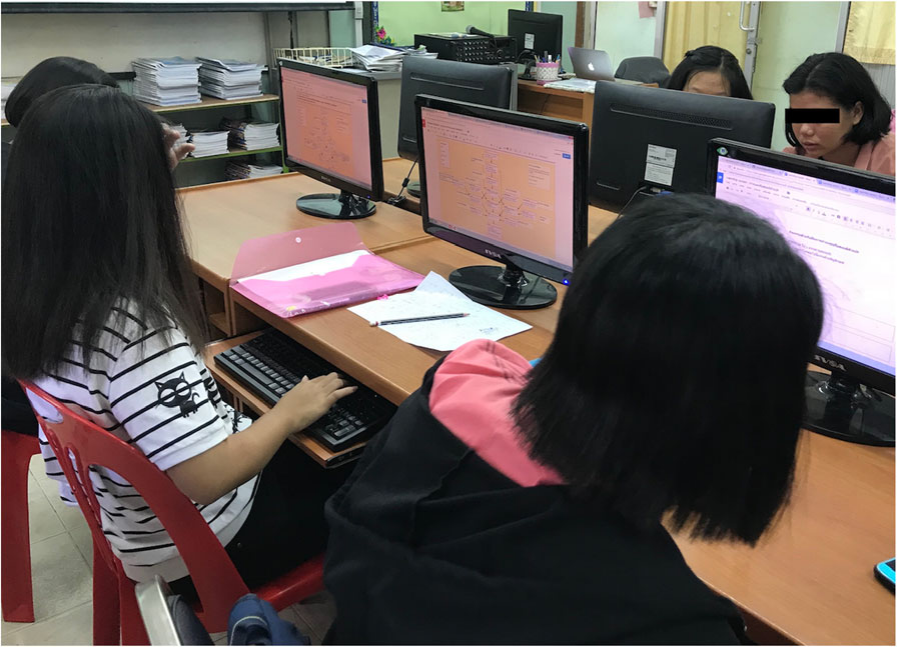
Learning environment of CREMA

In the following sections, we explain the procedure of the experimental design from sampling procedure to the methodology of data collection, in order to be able to answer our research questions.

### Sampling

The experiment was conducted at a public school in a small district in the northeast of Thailand in the province named Kalasin. Most students of this school come from the surrounding rural villages of the district where farming is practiced by the majority of the population, i.e. parents of the most students are farmers. To differentiate students who gained improvement by the training from those who had already been self-regulated learners prior to the training, we specifically considered students who were confused and could not recognize/realize their difficulties in solving MWP. The subjects were sampled from grade 9 students. The grade 9 students in this school had already learnt MWP when they were in grades 7 and 8. First, we selected 7 out of a total of 12 classes of the grade 9 students in the school based on their teachers’ report that the students of these 7 classes were low performance students with comparable mean socio-economic status level. Then, these 7 classes of students were screened into 3 classes by a MWP solving test together with metacognitive questions. Its example is shown as follows.

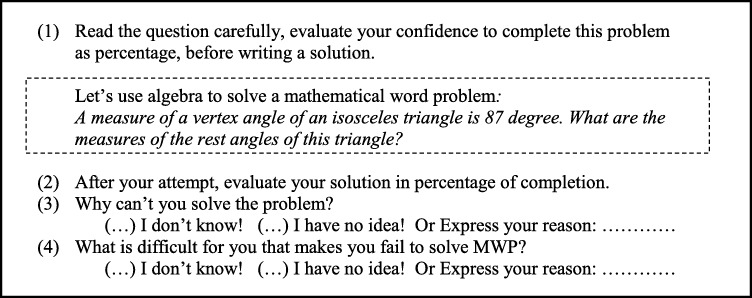



In addition, a result from Q-L2SRL was also considered in this screening. The detailed explanation of Q-L2SRL is explained in the section “Data collection instruments”. This screening process was taken about 1 month before the intervention. We selected 3 classes of students based on the MWP failing rate of each class and the students’ inability to express their reasons in the metacognitive questions and based on the fact that their Q-L2SRL pretest must not be significantly different.

### Teaching and learning procedure

The three selected classes from the sampling process were assigned to the three distinct learning groups (i.e., **CTRL**, **CTRL+MetaQ**, and **CREMA**). The numbers of students in each group/class were 37 (17 males, 20 females), 37 (17 males, 20 females), and 36 (13 males, 23 females), respectively. All groups learnt the same MWP solving topic and experienced the same level of practice problems selected from the textbook they used in the school. The total course was composed of six periods (50 min each) spanning 3 weeks (two periods a week). The learning procedure in each group is described as follows:

- **CTRL**: A mathematics teacher in the school taught the students in this group using a traditional method. She used white broad and explained how to solve MWP in front of the class. The teacher gave homework and assignments to the students after each class. The students’ works were checked as correct (checked mark) and incorrect (cross mark). The teacher often showed the solutions of some assignments in the beginning of her class and asked the students to take note.
- **CTRL+MetaQ**: The corresponding author (TD) taught students in this group by himself using the same traditional method and the same material as for the CTRL group. This is to control the way to deliver MetaQ. In this group what is different from the control group is that MetaQ’s were raised during the class while the author was giving lecture and during the time the students were practicing solving the problems. Moreover, the students’ assignments were returned with comments and suggestions about possibilities of their failures. The author taught the students himself to be able to manage how to provide MetaQ.
- **CREMA**: In this group, the students used computers as a medium to learn MWP—the learning procedure in this group is explained in section “CREMA”. The teacher of this group (the corresponding author: TD) monitored, controlled, and managed the atmosphere of the class. The teacher took responsibility as a facilitator and supporter when the students needed some help or were confused with the learning flow. The training program was composed of three phases inherited from CREMA: Preparation phase, Observation phase, and Experiencing phase, as explained in the section “CREMA”.

Please note that, due to time constraint of the experiment coupled with the tight schedule of the school curriculum, the same teacher could not be responsible for all three groups. To compare that each respective teacher conformed to the condition set for each group, TD and the schoolteacher had discussed what to be taught and how much explanation was allowed to be provided for the same mathematical problems prior to the start of the experiment. Also, the teaching of all three classes was based on the same material.

### Data collection instruments

To perform pretest and posttest evaluation, MWP test and Q-L2SRL were conducted before and after the intervention. The intervention was taken place about 1 month after conducting the pretest.

#### MWP test

MWP test was applied with metacognitive questions as shown in section “Sampling”, to investigate the students’ performance that clarified their self-difficulties in solving MWP. The posttest was composed of 6 MWPs. The problems were selected and modified from the student textbook that they normally used in the school. The English translations of all six problems of the posttest are shown in Appendix 3.

#### Q-L2SRL

Q-L2SRL has been especially developed for this research. It was modified from the items in Table 1 by separating each item into UL and ASL. As a result, there were 16 items in Q-L2SRL; see its English translation in Table 3. The questionnaire was composed in 4-point Likert-type scale (1 = I do not agree at all, 2 = I do not agree, 3 = I agree, and 4 = I strongly agree) allowing the students to express their consensus how much they agreed or disagreed with a particular statement. A reliability analysis was carried out on Q-L2SRL comprising all 16 items. Cronbach’s alpha showed the questionnaire has good internal consistency (*α* = 0.95). All items appear to be worthy of retention, resulting in a decrease in the alpha if deleted. Moreover, it also has adequate test-retest reliability (*r*(43) > 0.85, *p* < 0.0001 over a 3-week period) for all 16 items.

**Table 3:** Items of the Q-L2SRL and their Pearson’s correlation values from the test-retest reliability

Item codes	Questionnaire items	*r*	*p* value
UL-STM-A	I am (began to be)* curious about the source of my feeling to learn MWP.	0.896	0.000
UL-STM-G	I am (began to be) curious about my goal of MWP learning.	0.971	0.000
UL-STM-M	I am (began to be) curious about my reason why I should have to learn MWP.	0.943	0.000
UL-SUT-K	I am (began to be) curious about what I know in learning MWP.	0.954	0.000
UL-SUT-P	I am (began to be) curious about my understanding of MWP principle.	0.954	0.000
UL-SUT-D	I am curious (began to be) about my difficulty in MWP learning.	0.960	0.000
UL-SUP-S	I am (began to be) curious about the appropriate strategy to achieve my goal in MWP learning.	0.931	0.000
UL-SUP-C	I am (began to be) curious about the source of my distraction in learning MWP.	0.886	0.000
ASL-STM-A	I am (began to be) curious to find the benefit/application of learning MWP to make it easy for me to learn MWP.	0.870	0.000
ASL-STM-G	I am (began to be) curious about how to encourage myself to achieve the goal I set for learning MWP.	0.908	0.000
ASL-STM-M	I am (began to be) curious about the reason why I should have to learn MWP.	0.902	0.000
ASL-SUT-K	I am (began to be) curious about how to update my background knowledge to meet the knowledge required for learning MWP.	0.850	0.000
ASL-SUT-P	I am (began to be) curious about finding method to improve my understanding of MWP principle.	0.951	0.000
ASL-SUT-D	I am (began to be) curious about finding the way to resolve my difficulty in MWP learning to be able to improve my performance.	0.947	0.000
ASL-SUP-S	I am (began to be) curious about finding my own effective strategy to achieve my goal in MWP learning.	0.881	0.000
ASL-SUP-C	I am (began to be) curious about how to concentrate on the process during learning MWP.	0.869	0.000

Correlation (*r*) and significance level (*p*)

*The phrase in the parenthesis is used for the posttest

## Experimental result and analysis

There were some students who were not able to attend all sessions of the class reducing the total numbers of students in each class to 33 (13 male, 20 female), 34 (16 male, 18 female), and 34 (12 male, 22 female) in the **CTRL**, **CTRL+MetaQ**, and **CREMA** groups, respectively.

Table 4 shows descriptive statistics and ANOVA results of the Q-L2SRL posttest. The groups did not differ in the Q-L2SRL pretest (most students had no S2SRL in MWP learning), but in the Q-L2SRL posttest by performing a one-way ANOVA, a significant difference was found (*F*(2,98) = 128.05; *p* < .001), which may be ascribed to the intervention. A Tukey post hoc test revealed that students in **CREMA** (*M* = 3.58, *SD* = .43) were found to benefit the most from the intervention, gaining significantly higher S2SRL in MWP learning than the other two groups, and S2SRL in MWP learning in students of the **CTRL+MetaQ** group (*M* = 2.28, *SD* = .76) was significantly higher than that of the **CTRL** group (*M* = 1.38, *SD* = .53).

**Table 4:** ANOVA results and descriptive statistics for Q-L2SRL posttest

					Tukey’s HSD comparisons	
	*N*	*M*	*SD*	*SE*	**CTRL**	**CTRL+MetaQ**
**CTRL**	33	1.38	0.53	0.09		
**CTRL+MetaQ**	34	2.28	0.76	0.13	< 0.001	
**CREMA**	34	3.58	0.43	0.07	< 0.001	< 0.001
Source		*df*	*SS*	*MS*	*F*	*P*
Between groups		2	81.56	40.79	128.05	0.000
Within groups		98	31.22	0.32		
Total		100	112.79			

To confirm the effect of the intervention is independent of the student initial status, an ANCOVA was performed controlling for the Q-L2SRL pretest (see Table 5). The results confirmed the finding that the intervention had a significant main effect to support students to gain S2SRL in MWP learning (*F*(2,97) = 127.13, *p* < .001), and there was no effect from their initial status in these groups of students.

**Table 5:** ANCOVA results, multiple comparisons, and mean differences in Q-L2SRL posttest for Q-L2SRL pretest

Source	*df*	*SS*	*MS*	*F*	*P*
Q-L2SRL pretest	1	.33	.33	1.03	.31
Groups	2	80.97	40.48	127.13	.00
Error	97	30.89	.32		
Comparisons		Mean difference	*SE*	*P*	Bonferroni Adjusted 95% CI
**CREMA** vs **CNTRL**		2.20*	.138	<.001	1.86, 2.53
**CREMA** vs **CNTRL+MetaQ**		1.18*	.137	<.001	.85, 1.52
**CNTRL+MetaQ** vs **CNTRL**		1.01*	.138	<.001	.68, 1.35

*R*^2^ = .73, Adj. *R*^2^ = .72

Comparisons based upon ANCOVA adjusted means controlling for Q-L2SRL-pretest mean of 1.1108

* p < .05, where p-values are adjusted using the Bonferroni method

To consider the intervention more in detail, the responses to the individual items of Q-L2SRL are considered (Table 6). In traditional instruction (**CTRL**), some students became curious on their self-understanding and their self-improvement of self-understanding toward task (i.e., UL-SUT-K, UL-SUT-P, UL-SUT-D, ASL-SUT-K, ASL-SUT-P, and ASLSUP-S). Applying MetaQ in class **CTRL+MetaQ** could encourage more students to become curious about their self-understanding and their self-improvement of self-understanding toward task and in the other aspects. By delivering MetaQ adaptively with various kinds of support (**CREMA**), we could encourage a greater number of students to become curious about all of the required aspects.

**Table 6:** Frequencies of students who gained S2SRL in MWP learning for individual items

Item codes	Q-L2SRL Pretest			Q-L2SRL Posttest		
	**CTRL**	**CTRL+MetaQ**	**CREMA**	**CTRL**	**CTRL+MetaQ**	**CREMA**
	*N =* 33 (%)	*N =* 34 (%)	*N =* 34 (%)	*N =* 33 (%)	*N =* 34 (%)	*N =* 34 (%)
UL-STM-A	0	0	0	0	53	85
UL-STM-G	0	0	0	0	62	97
UL-STM-M	3	3	0	6	71	94
UL-SUT-K	3	0	3	27	74	97
UL-SUT-P	3	6	6	24	76	97
UL-SUT-D	3	0	3	33	74	97
UL-SUP-S	3	0	3	9	71	97
UL-SUP-C	0	0	0	0	74	97
ASL-STM-A	0	0	0	0	41	91
ASL-STM-G	0	0	0	0	35	97
ASL-STM-M	0	0	0	0	32	94
ASL-SUT-K	0	0	0	30	38	97
ASL-SUT-P	0	0	0	27	35	97
ASL-SUT-D	0	0	0	6	26	97
ASL-SUP-S	0	0	0	24	38	97
ASL-SUP-C	0	0	0	3	35	88

Table 7 compares the differences of frequencies of students who could specifically express their difficulties in solving MWP in the pretest and posttest against the three groups. All students in **CREMA** could state their difficulties and reasons why they failed to solve the problems. About 32% of the **CTRL+MetaQ** students could express their difficulties and none of the students in the **CTRL** group could do this task (e.g., students only wrote: I do not understand, I cannot remember, It is too difficult, or left it blank).

**Table 7:** Differences of frequencies of students who specifically expressed their difficulties in solving MWP in the pretest and posttest of MWP among the three groups

	*N*	Number of students who can express their difficulties	
		MWP-Pretest (%)	MWP-Posttest (%)
**CTRL**	33	0	0
**CTRL+MetaQ**	34	0	32
**CREMA**	34	0	100

We also consider the effect of the intervention on the students’ MWP solving proficiencies. All students failed in the MWP pretest. They had no difference in the MWP pretest. By performing a one-way ANOVA in the MWP posttest, a significant difference was found (*F*(2,98) = 4.08; *p* = .01) (Table 8). This may also be attributed to the intervention. A Tukey post hoc test revealed that students in **CREMA** (*M* = 15.12, *SD* = 8.28) performed significantly better in the MWP posttest than students in **CTRL** (*M* = 10.18, *SD* = 5.10), while students in **CTRL+MetaQ** (*M* = 12.62, *SD* = 5.70) also scored higher in the MWP posttest than those in **CTRL** albeit without statistical significance.

**Table 8:** ANOVA results and descriptive statistics for MWP posttest

					Tukey’s HSD comparisons	
	*N*	*M*	*SD*	*SE*	**CTRL**	**CTRL+MetaQ**
**CTRL**	33	10.18	5.10	0.89		
**CTRL+MetaQ**	34	12.62	5.70	0.98	0.28	
**CREMA**	34	15.12	8.28	1.42	< 0.01	0.26
Source		*df*	*SS*	*MS*	*F*	*P*
Between groups		2	408.09	204.04	4.08	0.01
Within groups		98	4168.47	42.54		
Total		100	4576.55			

To confirm the effect of the intervention is independent of the MWP pretest score, an ANCOVA was performed controlling for the MWP pretest (see Table 9). The results confirmed that the intervention had a significant effect on MWP solving performance (*F*(2,97) = 4.87, *p* = .01), and there was no effect from their MWP pretest.

**Table 9:** ANCOVA results, multiple comparisons, and mean differences in MWP posttest for MWP pretest

Source	*df*	*SS*	*MS*	*F*	*P*
MWP Pretest	1	50.46	50.46	1.19	0.28
Groups	2	413.06	206.53	4.87	0.01
Error	97	4118.01	42.45		
Comparisons		Mean Difference	*SE*	*P*	Bonferroni Adjusted 95% CI
**CREMA** vs **CNTRL**		4.93	1.13	< .01	7.92, 12.42
**CREMA** vs **CNTRL+MetaQ**		2.50	1.12	.39	10.39, 14.83
**CNTRL+MetaQ** vs **CNTRL**		2.44	1.12	.39	12.92, 17.36

*R*^2^ = .10, Adj. *R*^2^ = .07

Comparisons based upon ANCOVA adjusted means controlling for Q-L2SRL-pretest mean of 1.02

* *p* < .05, where p-values are adjusted using the Bonferroni method

Moreover, we found that there is positive correlation between the MWP-posttest score and the Q-L2SRL posttest score, *r*(101) = 0.34, *p* < 0.01.

## Discussion and conclusion

In this paper, we proposed a terminology, S2SRL, as a skill in which learners are curious about their own understanding and are aware of their self-improvement in their learning before they can perform metacognitive questions by themselves to reflect on their own cognition for planning, monitoring, and doing self-evaluation. Then, we precisely defined S2SRL in MWP learning for applying as a framework to evaluate our proposed model, CREMA, which is developed as a framework to design a learning environment encouraging learners to use intrinsic comprehension of metacognitive questioning to acquire S2SRL in MWP learning. Here, we addressed the following questions to assess our proposed framework: (i) Can CREMA really support learner to gain S2SRL and (ii) how does it work in a practical environment? In the second question, we considered it in two points: Is MetaQ a factor in CREMA to support learners to gain S2SRL? And can computer support really enhance training effect in CREMA?

To answer the first question, the questionnaire, Q-L2SRL, was developed to assess whether students have gained S2SRL in MWP learning, i.e., whether they began to be curious about their own understanding and were aware of their self-improvement in MWP learning having trained in the environment influenced by our proposed model, CREMA. The questionnaire, Q-L2SRL, was applied on the class of students who learnt MWP with our proposed method by implementing CREMA (**CREMA**) and the class of students who learnt MWP solving in traditional method (**CTRL**). The result revealed that our proposed model, CREMA, is effective for encouraging students to become curious about their own understanding and become better aware of their self-improvement in MWP learning for all considered aspects in Table 2.

To answer our second question, first, we needed to evaluate the effectiveness of MetaQ, which refers to metacognitive questions and motivational statements. This was an important step because MetaQ is an integral element in all phases of CREMA and has served the central role in our proposed model. There have been studies, which show benefits of training learning skills using metacognitive questions and answers (Jacobse and Harskamp 2009; Mevarech and Kramarski 2003). To confirm the effectiveness of MetaQ, the performance of students in the class where MWP solving was learned in a traditional way and was coupled with MetaQ (**CTRL+MetaQ**) was compared against that of the **CTRL** group. The result showed that MetaQ was a factor affecting students to gain S2SRL in MWP learning. However, due to our limitation in terms of instructor, we could not rule out the possibility of having different instructors partly contributing to the differences in the performance of the students after the experiment.

Secondly, we postulated that computer technology could be another contributing factor that enhances students’ learning sense empowering methodology to facilitate learning objects, in CREMA. The comparison between **CTRL+MetaQ** and **CREMA** could be used to validate our postulation. From our class observation, we can demonstrate our claim that students in **CREMA** individually received MetaQ related with what they were focusing in and they had equal chance to respond to MetaQ and got suggestion related with their behaviors from the system. However, those in the **CTRL+MetaQ** group, despite receiving the same MetaQ delivered by their teacher, their responses to the MetaQ varied—some did think about the MetaQ but others played with their friends and chose not to listen to the teacher. Due to the high number of students, the teacher could not take care of individual students effectively. This can be an explanation why the frequencies of positive responding students in **CREMA** were greater than those in **CTRL+MetaQ** and the means of Q-L2SRL of students in **CREMA** are significantly greater than of the students in **CTRL+MetaQ**.

In addition, all students in **CREMA** could state their difficulties and reasons why they failed to solve the problems in the MWP posttest, which was in great contrast to students in the other groups. This was evidence showing that they gained a basis skill to clarify their self-difficulties, which may be used to develop their MWP learning performance. Only in **CREMA**, QAS and InDi were applied as a representation to support students to gain more understanding in MWP solving process and to help them clarify their self-difficulties in the tasks, which would eventually help them to set their sub-learning goal to fulfill their difficulties. This was another way to support students in **CREMA** in order that they were able to precisely state their difficulties in problems they failed to solve. Moreover, the students in **CREMA** were outstanding from the other groups, especially, in the comparison with **CTRL** and there was a positive correlation between their MWP posttest score and their Q-L2SRL posttest score.

In conclusion, the implementation of our proposed model, CREMA, could effectively support learners to gain S2SRL in MWP learning, in which MetaQ played a key role in CREMA while appropriate emerging Optional supports (Explanation, think representation, practice) could enhance the effect of MetaQ. And by integrating MetaQ with computer and technology, it could enhance learners’ learning sense and to increase or expand the potential and efficiency of the use of learning objects, while MWP involves a process which benefits training metacognition in which we could use its benefit to prepare representation of learning process enabling the students interact with for example images and charts that would aid their understanding of the topics. Our finding reveals an alternative direction to design a meta-level thinking learning environment by defining the term, S2SRL, as a basis to develop our proposed framework, CREMA. We recognize the need to define and examine components of CREMA that are linked to qualities of mutual engagement and learners’ learning. Moreover, we recognize the need to understand more about how MetaQ is integrated with different kinds of support in different advanced technology environments. However, further research is needed to investigate the long-term effect of such support. It is interesting to compare a group of learners who have S2SRL against that of novices who are in the development process to become self-regulated learners. Equally interesting is what other kinds of support could be provided in the model to improve its effectiveness and to ensure that learners become more independent in learning and change their status from passive to active learners. Through our research, we hope that metacognition will become better recognized as a useful tool that helps students and learners alike to develop their own metacognitive techniques, which will enable them to tackle real-life problems in future.

## Appendix 1

### Stimulus

- Most interviewees expressed that they liked mathematics. Few students said mathematics was difficult; however, they thought the topic that they had to learn was basic mathematics necessary for their future. Although they could ignore it at the time, they still had to face it in the future. They did realize that it would make more sense make more effort to learn mathematics now than suffer the effect of missed opportunity in future.
- For the students who liked solving MWP, they would like to accomplish more advanced problems. They said they were very happy when they could solve difficult problems that other students could not. For the students who did not like mathematics, they would like to maintain their grade in a good level for their future, which would also make their parents happy.
- It is quite obvious that the students who liked mathematics had intrinsic motivation to accomplish this task. For students who did not like mathematics, upon struggling with difficult exercises, they were concerned about their future and what their family would say if the family had found out they were not good enough at mathematics. Their teacher was also another contributing factor pushing them to try harder.

### Self-understanding toward task

- Most students gave consistent statements and showed the evidence that they were curious about what they knew or did not know for the class. For the students who liked to solve MWP, they constantly searched for more challenging problems and pondered about their difficulties and yet tried to find a way to solve them. They though it was very important for them to overcome their weak points and improve themselves.
- Most students agreed that being able to have a clear understanding of the problem structure and its principle could help them solve unseen problems better.
- Understanding of their own difficulties was an important aspect that most students mentioned. The students stated that when they could not solve some problems, they asked their more able friends or the teacher to help explain points that lead to them struggling with the problem. And to make sure that they could really overcome their weak points, it was important to for them to have clarification on those points and then they would subject themselves to similar problems really ensuring that they had those weak points behind them.

### Self-understanding toward learning process

- Most students always reflected whether they did well in learning MWP, whether they were still on the way to achieve good score in the class, whether what they did during the classes really helped them to keep their good progress, and whether there was anything they had to change so their performance could be improved.
- For some students who liked mathematics, they rarely had distraction during their classes. They happily learnt and practiced MWP. The students who did not like mathematics expressed an interesting point – because they realized they did not like the topics, they avoided getting to the state where confusion could occur, which might lead to them becoming bored and possibly failing to reach their goals.

## Appendix 2

**Table 10:** Comparison of two groups of participants who were confident as self-regulated learners (SR) and who were confident as non self-regulated learners (nonSR) for individual items in the third part of the questionnaire

Item codes	Participant groups	*N*	*M*	*SD*	*SE*
STM-A	SR	247	5.46	0.99	0.98
	notSR	125	2.76	1.46	2.12
STM-G	SR	247	5.34	1.14	1.31
	notSR	125	2.66	1.34	1.79
STM-M	SR	247	5.67	1.07	1.24
	notSR	125	2.69	1.62	2.62
SUT-K	SR	247	5.76	1.11	1.23
	notSR	125	3.25	1.34	1.8
SUT-P	SR	247	6.14	0.91	0.82
	notSR	125	3.95	1.31	1.71
SUT-D	SR	247	5.85	0.88	0.78
	notSR	125	3.57	1.50	2.26
SUP-S	SR	247	5.57	0.96	0.93
	notSR	125	3.66	1.23	1.52
SUP-C	SR	247	5.72	1.03	1.06
	SR	125	3.44	1.42	2.01

*t*_STM-A_ = 21.05391, (t_STM-A_ refers to *t* value for STM-A),

*t*_STM-G_ = 20.11888, *t*_STM-M_ = 21.25528, *t*_SUT-K_ = 19.19453, *t*_SUT-*P*_ = 19.19453,

*t*_SUT-D_ = 18.4523, *t*_SUP-S_ = 18.4523, *t*_SUP-C_ = 17.7273, for all *p* < 0.01

## Appendix 3

### The MWP Posttest

1. If the sum of a number and 231 is equal to 756, please find that number.
2. Mom gave Kapom a 1000Baht banknote to pay for the electricity bill. How much did he have to pay for the electricity, if Kapom received 121.50 Baht back after the payment?
3. A measure of a base angle of an isosceles triangle is 21 degree. What are the measures of the rest angles of this triangle?
4. The total of number of oranges and apples is 77. If the number of oranges is 13 more than the number of apples, please find the number of apples.
5. A collection of 155 coins, consisting of 1Baht coins and 5Baht coins, has a value of 395Baht. Please find how many coins of each kind there are
6. The sum of the ages of father and son is 83 and a mother is 42 years old. If 4 years ago the father’s age was twice that of his son, how old is the son now?

## Abbreviations

A: Attitude
ASL: Awareness of self-improvement in MWP learning
C: Learning concentration
CREMA: Computer-Supported Meta-Reflective Learning Model via MWP
**CREMA**: A group of students who learnt MWP solving via computer application implemented by applying CREMA as a framework
**CTRL**: A group of students who learnt MWP solving by traditional method
**CTRL+MetaQ**: A group of students who learnt MWP solving by traditional method combining with the intervention of MetaQ
D: Self-difficulty
G: Goal
InDi: Inferential diagram
K: Background knowledge
M: Motivation
MetaQ: Metacognitive questions and motivational statements
MWP: Mathematical Word Problem
P: Self-understanding of principle of a topic
PISA: Programme for International Student Assessment
QAS: Q/A sequence
S: Strategy
S2SRL: Seed skill TO become a Self-Regulated Learner
STM: Stimulus
SUP: Self-understanding toward learning process
SUT: Self-understanding toward task
TIMSS: Trends in International Mathematics and Science Study
UL: Understanding of MWP learning
